# Kinetics of the Gas-Phase Reaction of Hydroxyl Radicals with Dimethyl Methylphosphonate (DMMP) over an Extended Temperature Range (273–837 K)

**DOI:** 10.3390/molecules27072301

**Published:** 2022-04-01

**Authors:** Xiaokai Zhang, Daria A. Barkova, Pavel V. Koshlyakov, Ilya E. Gerasimov, Evgeni N. Chesnokov, Lev N. Krasnoperov

**Affiliations:** 1 Department of Chemistry and Environmental Science, New Jersey Institute of Technology, Newark, NJ 07102, USA; zhangxz458@njit.edu; 2 Institute of Chemical Kinetics and Combustion, Siberian Branch of Russian Academy of Sciences, 630090 Novosibirsk, Russia; d.plastinina@g.nsu.ru (D.A.B.); pvk@kinetics.nsc.ru (P.V.K.); gerasimov@kinetics.nsc.ru (I.E.G.), chesnok@kinetics.nsc.ru (E.N.C.)

**Keywords:** kinetics, hydroxyl, radical, dimethyl methylphosphonate, DMMP, laser photolysis, transient absorption, V-shaped temperature dependence, pressure dependence

## Abstract

The kinetics of the reaction of hydroxyl radical (OH) with dimethyl methylphosphonate (DMMP, (CH_3_O)_2_CH_3_PO) (reaction 1) OH + DMMP → products (1) was studied at the bath gas (He) pressure of 1 bar over the 295–837 K temperature range. Hydroxyl radicals were produced in the fast reaction of electronically excited oxygen atoms O(^1^D) with H_2_O. The time-resolved kinetic profiles of hydroxyl radicals were recorded via UV absorption at around 308 nm using a DC discharge H_2_O/Ar lamp. The reaction rate constant exhibits a pronounced V-shaped temperature dependence, negative in the low temperature range, 295–530 K (the rate constant decreases with temperature), and positive in the elevated temperature range, 530–837 K (the rate constant increases with temperature), with a turning point at 530 ± 10 K. The rate constant could not be adequately fitted with a standard 3-parameter modified Arrhenius expression. The data were fitted with a 5-parameter expression as: k_1_ = 2.19 × 10^−14^(T/298)^2.43^exp(15.02 kJ mol^−1^/RT) + 1.71 × 10^−10^exp(−26.51 kJ mol^−1^/RT) cm^3^molecule^−1^s^−1^ (295–837 K). In addition, a theoretically predicted pressure dependence for such reactions was experimentally observed for the first time.

## 1. Introduction

During the last decades, organophosphorus compounds have been recognized as efficient flame retardants even at relative low concentrations [1,2,3,4,5,6,7,8,9,10]. On the other hand, organophosphorus compounds also exist in the environment as pesticides, insecticides, etc. [11,12]. Detailed mechanisms of the flame retardancy as well of the destruction of these compounds in the environment require detailed information on the kinetics of the elementary reactions of these compounds in the reaction mechanisms. Currently, such data, even for the reactions with the major oxidation species, hydroxyl radical (OH), are very sparse. Specifically, for the target reaction of this study, reaction of hydroxyl radical with dimethyl methylphosphonate, DMMP, (CH_3_O)_2_CH_3_PO (Equation (1)), only a single indirect kinetic study exists [13].

OH + (CH_3_O)_2_CH_3_PO → products (1)

In this single gas phase indirect kinetic study the rate of reaction (1) was measured relative to the rate of reaction of hydroxyl radicals with n-butyl ether (n-C_4_H_9_)_2_O over the temperature range 283–348 K. A negative temperature dependence was observed in this temperature range (reaction slows down with temperature). Similar behavior was previously observed for the reaction of hydroxyl radical with trimethyl phosphate (TMP, (CH_3_O)_3_PO) [14], where the negative temperature dependence at low temperatures switched to a positive temperature dependence at elevated temperatures, resulting in a V-shaped temperature dependence. Such a behavior was predicted in the interpretation of the kinetics of the reaction CH_3_ + HBr [15,16] based on the concept of “negative” or “submerged” reaction barrier.

The reaction was also studied in solution using pulsed radiolysis; using relative rate technique, important mechanistic conclusions have been derived [17].

In this work, the kinetics of reaction (1) was investigated using pulsed laser photolysis combined with transient UV absorption over an extended temperature range (295–837 K). A peculiar V-shaped temperature dependence was observed similar to that previously reported for the reaction of OH with trimethyl phosphate (TMP) [14]. Pressure dependence was also observed in the pressure range 0–30 bar, as predicted in the theoretical study [16].

The measurements were performed in two laboratories, Institute of Chemical Kinetics and Combustion, ICKC, Novosibirsk, Russia and New Jersey Institute of Technology, NJIT, Newark, USA. In both studies hydroxyl radicals (OH) were formed using fast reaction of electronically excited atoms O(^1^D) with H_2_O molecules [18,19].

O(^1^D) + H_2_O → 2OH (2)

Reaction 2 is very fast, the reaction of O(^1^D) with H_2_O requires only a few collisions (the rate constant k_2a_ = 2.0 × 10^−10^ cm^3^molecule^−1^s^−1^ at 298 K) [19]. Moreover, due to the relatively strong O-H bond in the water molecule, reaction (2) produces fewer vibrational and rotational excitations, compared to other reactions with H-containing molecules. Moreover, reaction (2) produces two hydroxyl radicals per one excited oxygen atom, and no other free radical species, which can complicate the reaction mechanism. Therefore reaction (2) was chosen as the source of hydroxyl radicals in this study.

At the Institute of Chemical Kinetics and Combustion (ICKC), electronically excited oxygen atoms were produced by photolysis of ozone at 266 nm:

O_3_ + hν(266 nm) → O(^1^D) + O_2_ (3)

The temperature range of this study was 273–470 K, the highest temperature was limited by the thermal stability of ozone. The temperature range was significantly expanded using a different photolysis system, available at NJIT. Specifically, to produce excited oxygen atoms, photolysis of N_2_O at 193 nm (ArF excimer laser) was used. N_2_O has much better thermal stability, compared with that of ozone. The replacement of excited oxygen atom photochemical precursor, allowed raising the upper temperature of the available temperature range to 837 K.

N_2_O + hν(193 nm) → O(^1^D) + N_2_ (4)

## 2. Experimental

The experimental set-ups at the ICKC and NJIT used in the study are similar. Both are based on pulsed laser photolysis coupled with a time resolved transient absorption spectroscopy. The only major difference is in the different photolysis systems used to produce excited oxygen atoms, O(^1^D).

### 2.1. Experimental: ICKC

The experimental set-up at ICKC was described previously [14,20]. Ozone produced on-line using a corona discharge dielectric barrier ozonator is mixed with other reactants in a flow system. Gas mixture of 10% O_2_ in He was passed through the ozonator. The typical degree of conversion of O_2_ to O_3_ was about 7%. The ozone concentration in the reactor was determined accurately and directly in situ by absorbance at the 253 nm using a mercury resonance lamp using accurately known absorption coefficients. Ozone is photolyzed at 266 nm (4th harmonic of an Nd:YAG laser, Lotis Tii, model: LS-2137U). Temporal profiles of OH absorption of light produced by a DC discharge hydroxyl lamp (multiline around 308 nm) passed through a monochromator are digitized, accumulated and stored using a digital oscilloscope. The typical concentration of ozone was ca. 3.44 × 10^14^ molecule cm^−3^. Typical concentration of molecular oxygen, O_2_, was ca. 8.2 × 10^15^ molecule cm^−3^. Hydroxyl radicals were generated in the reaction of excited oxygen atoms O(^1^D) with water molecules (reaction (2)). Typical concentration of water molecules was ca. 2.5 × 10^16^ molecule cm^−3^.

Hydroxyl radicals were monitored via absorption of light generated by a low pressure DC discharge lamp in a H_2_O/Ar mixture. The OH lamp is a quartz tube, with two metal fittings at the ends. Argon, saturated with water vapor at ca. 1 bar pressure and ambient temperature, was pumped through the lamp at 40 Torr (5.33 kPa). DC voltage of 5 kV was applied to the metal fittings of the OH lamp via a ballast resistor, the electric current was 30 mA. The light emitted from the lamp passed through the reactor was focused on the entrance slit of a monochromator (Carl Zeiss, SPM2). The spectral width was set ca. 12 nm to select a bunch of ca. 20 emission lines of hydroxyl radical around 308 nm. The temperature and pressure dependences of the apparent absorption cross-section of hydroxyl radical obtained with such a lamp as well as the curves-of-growth were studied in detail in the experiments as well as via modeling in previous studies [21,22,23]. For the optical arrangement at ICKC, used in the current study, the low pressure low absorbance (<2%) experimentally determined apparent absorption cross section of OH was 7.2 × 10^−17^ cm^2^molecule^−1^ at 293 K.

A heated metal flow reactor, 10 cm long with an inner diameter of 8 mm with fused quartz windows, was used. Before entering the heated reactor, the flowing reaction mixture was preheated to the reactor temperature via placing the 2 m entering tube as well as the reactor in a common heater. The temperature of the reactor ranged from 273 to 473 K. The pressure in the cell was ca. 12–15 Torr (1600–2000 Pa) above ambient atmospheric pressure and was in the range 750–775 Torr (99.98–103.30 kPa).

The main bath gas (helium) flowrate was measured with an SMC PFMV510-1 flowmeter. The main flow rate was 1000 sccm. The flowrate of the O_2_/He mixture was measured before entering the ozonator by an MF Matheson 8141 flow meter; the flow rate was ca. 4 sccm. Measurements and control of small helium flows passing through a two-stage water saturator was done using a TylanFC 260 mass flow controller. This helium flow was about 44 sccm.

The reactant (dimethyl methylphosphonate, DMMP) was introduced using an Agilent chromatographic syringe with a volume of 25 μL driven by a stepper motor. DMMP was supplied to the evaporator, which in turn was purged with the main helium flow. The flow rate of liquid DMMP ranged from 0 to 0.46 µL/min which, after evaporation, corresponds to 0–0.11 sccm. The DMMP concentration in the reactor was varied from 0 to 2 × 10^15^ cm^−3^.

### 2.2. Reagents (ICKC)

The purity of the gases used in the work were: He > 99.995%, O_2_ grade pure > 99.95%, H_2_O was deionized, the resistance was 17 MOhm cm, and Ar > 99.999%. DMMP was purchased from Aldrich (purity >97%).

### 2.3. Experimental: NJIT

The experimental set-up used at NJIT is described in detail in previous publications [21,22,23,24,25,26]. Hydroxyl radicals were generated in pulsed photolysis of N_2_O in the presence of water vapor at 193.3 nm (ArF excimer laser). The importance of different channels of the photodissociation process (reaction (4)) as well as of the subsequent reaction of O(^1^D) with H_2_O (reaction (2)) are also discussed in detail in previous publications [20,21,22,23].

The kinetics of hydroxyl radical decay was monitored by absorption in the UV (multiline at ca. 308 nm using low pressure H_2_O/Ar DC discharge lamp). Before entering the reactor, the laser beam was formed (to provide uniformity) with two lenses. The beam uniformity across the reactor cross-section was ±7.3% from the mean value. The gas flow rates were controlled by mass flow controllers (Brooks, model 5850). The total flow rates of the reactant mixtures with helium were in the range 11–12 sccs. Additional flush flows to the reactor windows were 2 sccs. A precision syringe pump (Harvard Apparatus, model PHD 4400) was used to inject liquid water. Pure liquid DMMP was used via injection using another syringe pump. The injection was performed via corresponding capillary tubes through an evaporator (kept at 90 °C) and, subsequently, to the reactor. In this way, steady flows of H_2_O and DMMP vapor were achieved. The total flow rate of these two liquids were kept constant at each given temperature, ranges from 6 to 11 μL/min, provided approximately the same concentration of H_2_O. The equivalent gas flow rate of DMMP in standard cubic centimeters per minute (sccm) was calculated based on the volumetric flowrate of the solution, based on the ideal gas law.

The concentrations of the precursors used were (1.94–3.24) × 10^17^ (H_2_O), (3.23–7.70) × 10^16^ (N_2_O) and (0–10) × 10^14^ (DMMP) molecule cm^−3^.

The absolute concentrations of OH radicals were determined based on the photon flux inside the reactor, the absorption cross-section of N_2_O at 193.3 nm and the efficiency of conversion of O(^1^D) atoms produced in the photolysis of N_2_O to OH radicals. The absorption cross-section of N_2_O is accurately known at 298 and 1 bar, at other conditions, the cross-sections of N_2_O were measured in previous works [20,21,22,23]. The model used for the calculations of the efficiency of conversion of excited O(^1^D) atom to OH radicals as well as the in situ laser light actinometry are described in detail in previous studies. The approach is based on the monitoring of ozone formation at 253.6 nm in the photolysis of N_2_O/O_2_/N_2_ mixtures at 1 bar and 298 K. The photolysis laser photon fluence inside the reactor was varied in the range of (4–9) × 10^15^ photon cm^−2^pulse^−1^. The initial concentrations of hydroxyl radicals were in the range (1.4–2.7) × 10^13^ molecule cm^−3^. All experiments were performed at 1 bar (He) pressure over 295–837 K temperature range.

### 2.4. Reagents (NJIT)

Helium used in the experiments was BIP Helium from Airgas with 99.9999% purity with reduced oxygen content (<10 ppb). UHP oxygen was obtained from Matheson TriGas (99.98% purity). Certified mixture of N_2_O in He (2.50%, accuracy ±2%) obtained from Matheson Tri-Gas was used. Purified water (Milli-Q) with TOC less than 5 ppb) was degassed by freeze-pump-thaw cycles and used as a reactant supplied by a syringe pump (Harvard Apparatus PHD 4400) as well as in the low pressure H_2_O/Ar discharge hydroxyl monitoring lamp. UHP argon obtained from Matheson TriGas (99.999% purity) was used in the H_2_O/Ar lamp. DMMP was purchased from Aldrich (purity >98%).

## 3. Results

**Figure 1:**
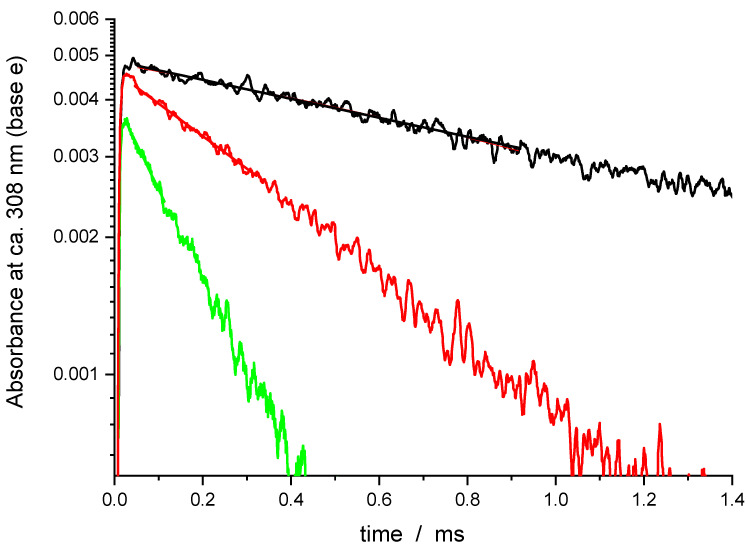
Sample transient absorption signals of OH in a O_3_/O_2_/H_2_O/He mixture at 266 nm and T = 411 K. [O_3_] = 3.43 × 10^14^, [H_2_O] = 2.3 × 10^16^, [He] = 1.8 × 10^19^. Black—without DMMP, red—[DMMP] = 3.67 × 10^14^ molecule cm^−3^, green—[DMMP] = 10.9 × 10^14^ molecule cm^−3^. Solid lines—exponential fits of ca. 1/3 of the initial amplitude (see text).

**Figure 2:**
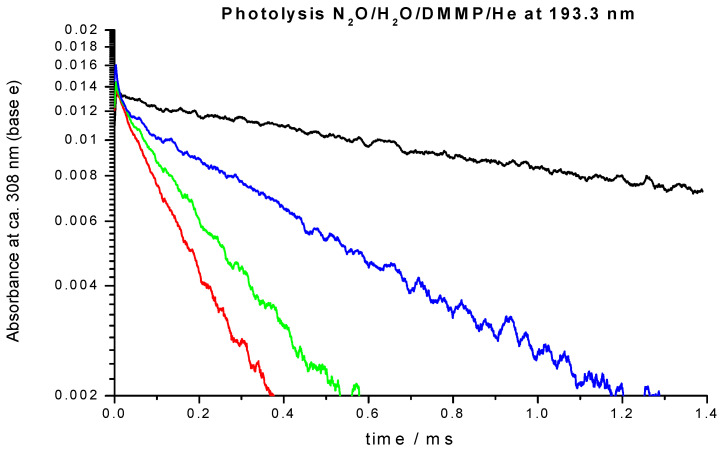
Sample UV absorption profiles of OH radical (multiline at ca. 308 nm). Photolysis of N_2_O/H_2_O/DMMP/He mixtures at 193.3 nm. T = 365 K, p = 1 bar. [N_2_O] = 4.49 × 10^16^ molecule cm^−3^, [H_2_O] = 2.91 × 10^17^ molecule cm^−3^, [N_2_O]/[H_2_O] = 0.154. Black—[DMMP] = 0, blue—[DMMP] = 1.92 × 10^14^ molecule cm^−3^, green—[DMMP] = 5.76 × 10^14^ molecule cm^−3^, and red—[DMMP] = 9.59 × 10^14^ molecule cm^−3^.

Sample OH absorption profiles obtained in the O_3_/O_2_/H_2_O/DMMP/He + 266 nm and N_2_O/H_2_O/DMMP/He +193 nm are shown in Figure 1 and Figure 2, respectively. The increase of the absorption in the figures corresponds to the formation of hydroxyl in reaction (2). The reaction of O(^1^D) atoms with H_2_O is extremely fast (the rate constant, k = 1.8 × 10^−10^ cm^3^/s) [18,19], therefore the conversion time of excited oxygen atoms did not exceed 1 μs.

Decay of the OH concentration is due to the different reactions of OH, including OH radical self-reaction, decay on the walls as well as the target reaction with DMMP. The secondary reactions of the products of reaction (1) might also contribute to the decay rates. In contrast to our previous studies of elementary reactions of hydroxyl radical and other radical species, where a system of differential equations that corresponds to the reaction mechanism was numerically solved and the resulting profiles were fitted to the experimental, a simplified analysis was accepted in this work. For reaction (1), currently no information about the products, branching ratios and the rate constants of subsequent reactions of the products of reaction (1), is available, and the previously used rigorous approach is not feasible. In lieu of the lack of a detailed reaction mechanism, no integrated kinetic decay curves could be obtained. Therefore, the method of the initial rates also widely used in chemical kinetics was applied as described below.

After formation of hydroxyl radicals in the reaction of excited oxygen atoms with water molecules, the radicals are consumed in the target reaction (1) as well as in the side reactions discussed above. Therefore, at short times the rate constant of consumption, k’, is written as:

(5) $$k^{\prime }={\left(-\frac{1}{{\left[\mathrm{OH}\right]}_{0}}\frac{d\left[\mathrm{OH}\right]}{\mathrm{dt}}\right)}_{t=0}$$

(6) $$k^{\prime }={k_{1}}^{\prime }+k_{0}$$

(7) $${k_{1}}^{\prime }={\;k}_{1}\left[\mathrm{DMMP}\right]$$

where k’ is the “initial slope rate constant” defined by Equation (5), k_1_′ = k_1_[DMMP] is the pseudo-first order rate constant of the target reaction, and k_0_ accounts for all other processes. In the evaluation of the initial slopes, the initial fractions of the decay curves should be properly fitted. There are numerous functions, including just the linear one, which can be used for this purpose. In the case of pseudo-first order decays, the decay profiles are exponential. Therefore, the exponential decay function was chosen to evaluate the initial decay rates, since it provides unbiased estimation of the “initial slopes” for pseudo-first order processes.

To evaluate the “initial slope” rate constant, the initial portion of the decay profiles (about 1/3 of the amplitude) was fitted with exponential function

[OH] = [OH]_0_ exp(-k’t) (8)

Then plotting the “initial slope rate constant” k’ vs. the reactant concentration [DMMP] one expects to obtain a straight line whose slope yields the rate constant of the target reaction (1), and the intercept the contribution of other processes (such as self-reaction) at the initial stage of the reaction.

The rate constant of the self-reaction of hydroxyl radicals is well characterized [20,21,22], and its contribution into the initial decay is easy assessable. Assessment of the potential interference of the reactions with the products of the target reaction (1) is not straightforward. The only possibility is to use very low initial concentrations of OH, to reduce the possible role of such processes. The maximum values of k’ were about 10,000 s^−1^. The majority of the experiments were performed with the initial absorbance of OH radicals not exceeding 1.5%, which translates to the initial concentrations of the radicals of ca. 2 × 10^13^ molecule cm^−3^. Assuming that the rate constants of the secondary reactions of OH with the presumable free radical products of reaction (1) do not exceed a typical value for barrierless radical-radical reactions of 3 × 10^−11^ cm^3^molecule^−1^s^−1^, such initial concentrations might contribute up to ca 3 × 10^−11^ × 2 × 10^13^ = 600 s^−1^, i.e., ca 6% of the maximum decay rate. Only ca. 1/3 of the initial amplitude was used in the decay fits to evaluate the “initial slope” rate constant, so that the above estimate of the contribution of the reactions with the reaction products is reduced further by a factor of 3, the contribution does not exceed 2% of the maximum rate.

**Figure 3:**
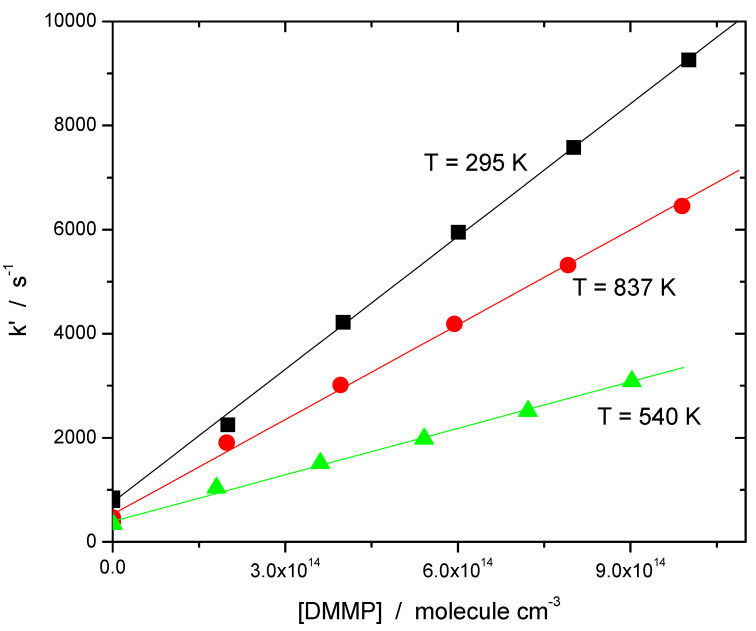
Sample dependences of the “initial slope rate constant, k’ ” on concentration of DMMP at three temperatures (low, high and an intermediate). Photolysis of N_2_O/H_2_O/DMMP/He mixtures at 193 nm. The slopes yield the rate constants of the target reaction (1). Negative temperature dependence (decrease of the rate constant with temperature) at low temperatures as well as positive temperature dependence (increase of the rate constant with temperature) at high temperatures are apparent.

Sample dependences of the “initial slope rate constant” k’, on the reactant (DMMP) concentration are shown in Figure 3. The dependences are linear, the slopes yield the rate constants of the target reaction (1). The negative temperature dependence (the decrease of the reaction rate with temperature, negative apparent activation energy) is apparent in the low temperature range. In the high temperature range, the rate constant increases with temperature (positive temperature dependence, positive apparent activation energy).

**Figure 4:**
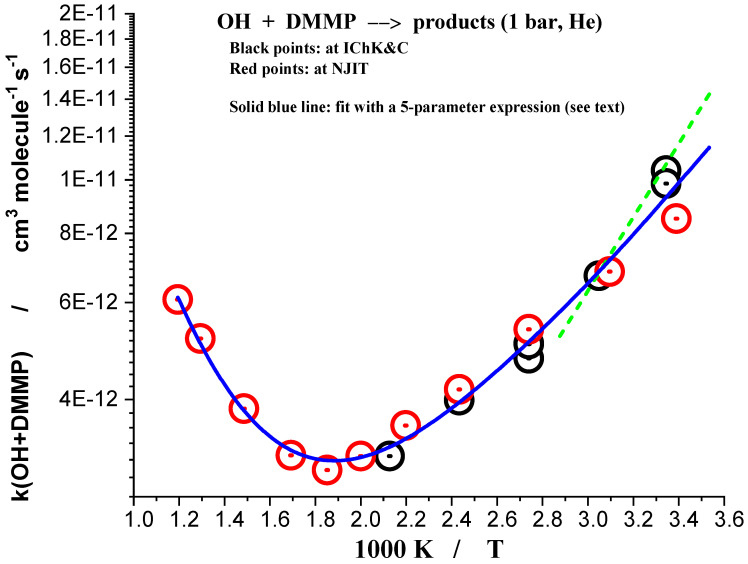
Summary. Rate constant of reaction (1) at 1 bar, over the temperature range 298–837 K. Red points: N_2_O/H_2_O/He + 193 nm, (at NJIT), black points: O_3_/H_2_O/He + 266 nm, (at ICKC). Blue line—fit by a 5-parameter expression (see text). Green dotted line: the only previous indirect experimental determination (relative rates method [13]).

The results of all measurements where ozone was used as photolytic source of excited oxygen atoms are listed in Table 1. The negative temperature dependence in the low temperature range is apparent. As it was already mentioned, the temperature range of the study was significantly extended by using N_2_O as a photolytic source of excited oxygen atoms, O(^1^D). N_2_O has much better thermal stability, thus the upper temperature of the experiments was raised to 837 K. Sample decay profiles obtained in the photolysis system N_2_O/H_2_O/TMP/He + hν(193 nm) at 365 K at several concentrations of the reactant, DMMP, are shown in Figure 2. Sample data processing via plotting the “initial slope” rate constant k’ vs. concentration of the reactant, TMP, is shown in Figure 3. The negative temperature dependence at low temperatures turns to a positive dependence at higher temperatures. All the results together with the experimental conditions are summarized in Table 1 and Table 2 and are shown in Figure 4 together with the results of the only previous (indirect) study [13].

The rate constant could not be adequately fitted with a standard 3-parameter modified Arrhenius expression. To provide an accurate fit to the experimental data, a minimum of five parameters are required. The data were fitted with a 5-parameter expression as:

k_1_ = 2.19 × 10^−14^(T/298)^2.43^exp(15.02 kJ mol^−1^/RT) + 1.71 × 10^−10^exp(−26.51 kJ mol^−1^/RT) (295–837 K) (9)

The modified transition state theory (MTST) that describes application of the concept of the transition state to the reactions where the ground state of the transition state lies below the ground state of the reactants (“negative barrier”, “submerged barrier”) explains not only the negative temperature dependence of the reaction rate at low temperatures, but also predicts the turning point in the temperature dependence (“V-shape”) and possible pressure dependence [16,26]. The theory predicts lower rate constant at low pressures, k_MTST_, compared to a larger rate constant at high pressures, k_TST_. The transition is expected at high pressures (tens and hundreds of bar), the impact of pressure is expected to be larger at low temperatures.

The V-shaped temperature dependence was observed in this study (OH + dimethyl methylphosphonate) as well as in the previous study, OH + trimethyl phosphate [14]. Similar dependences are expected for other organophosphorus compounds containing methoxy groups.

**Figure 5:**
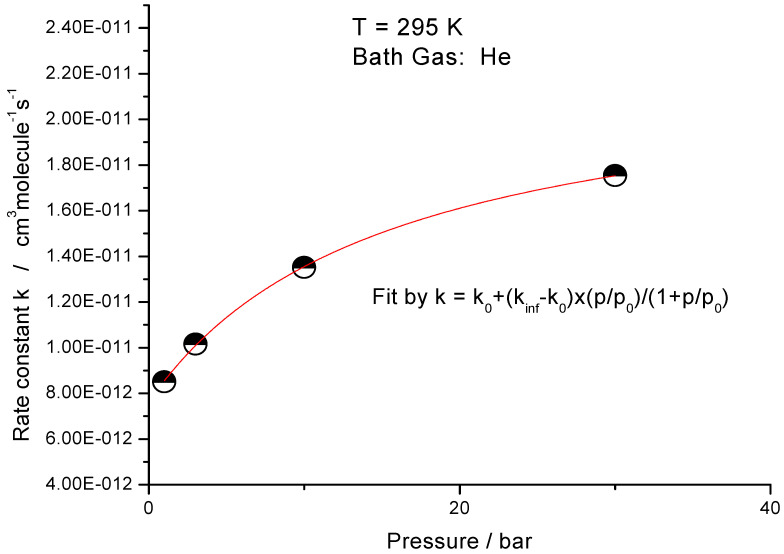
Pressure dependence of the rate constant of reaction (1). Bath gas—He. Temperature 295 K. Smooth curve—fit using a simple model (see text). Fitted parameters: k_0_ = (7.64 ± 0.12) × 10^−12^ cm^3^molecule^−1^s^−1^, k_inf_ = (2.25 ± 0.044) × 10^−11^ cm^3^molecule^−1^s^−1^, and p_0_ = (15.2 ± 1.2) bar.

However, such pressure effect on a rate of a “simple metathesis reaction” was not yet observed. We performed pressure dependent measurements over the pressure range 1–30 bar at the lowest temperature, currently available (ambient). The results are shown in Figure 5. The rate constant increases with pressure from 0.87 × 10^−11^ at 1 bar to 1.77 × 10^−11^ cm^3^molecule^−1^s^−1^ at 30 bar.

The data were fitted using the following expression:

k = k_0_ + (k_inf_ − k_0_)(p/p_0_)/(1 + p/p_0_) (10)

The expression (10) corresponds to a simple model, where all relaxation processes are described by a single parameter, p_0_, the transition pressure. The results of the fit are: the low-pressure limit rate constant, k_0_ = k_MTST_ = (7.64 ± 0.12)10^−12^ cm^3^molecule^−1^s^−1^, the high-pressure limit rate constant, k_inf_ = k_TST_ = (2.25 ± 0.05)10^−11^ cm^3^molecule^−1^s^−1^, and the transition pressure p_0_ = (15.2 ± 1.2) bar. The study is planned to be extended to a wider pressure range (0–100 bar) as well to lower temperatures (down to ca. −80 °C).

## 4. Discussion

The most prominent feature is the V-shaped temperature dependence of the rate constant—negative at low temperatures, positive at high temperatures, with the turning point at 530 ± 10 K. There could potentially be two reasons for such peculiar temperature dependence [14]. One possibility is that the ground state of the transition state lies below the ground state of the reactants (“negative barrier”, “submerged barrier” [16,26]. In such a case the modified transition state theory predicts V-shaped temperature dependence, negative at low temperatures, and positive at high temperatures, with possible pressure dependence at high pressures [16,26]. Another possibility is that the reaction has two or more channels, like direct H-atom abstraction from methoxy CH_3_-O- groups, methyl group CH_3_- as well as attachment of OH to the double P=O bond in DMMP:

OH + (CH_3_O)_2_CH_3_PO → H_2_O + CH_2_O(CH_3_O)CH_3_PO (11)

→ H_2_O + CH_2_(CH_3_O)_2_PO (12)

OH + (CH_3_O)_2_CH_3_PO → (CH_3_O)_2_CH_3_P(OH)O → products (13)

Neither theoretical works on the abstraction channels (11) and (12), nor any theoretical works on the prospective complex forming channel (13) with further transformations within the complex are currently available. Complex forming reactions often have negative temperature dependence; typical H-atom abstraction reactions usually have small positive barriers [27]. Theoretical calculations for the reaction of OH with trimethyl phosphate resulted in the “negative barriers” for several conformers of the transition state of the channel of H-atom abstractions from methoxy groups [28]. The analysis of the reaction products in the study in solutions by pulsed radiolysis [17] led to the conclusion that both the H-atom abstraction from the methyl group, channel (12), and attachment to the double bond, channel (13), are not important, thus that the only important channel is abstraction of H-atom from methoxy groups. This channel energetically should be similar to that in the reaction of OH with trimethyl phosphate, and is expected to have a submerged barrier. The experimental observations of this work (V-shaped temperature dependence and pressure dependence) support such an assumption.

## 5. Conclusions

The kinetics of the reaction of hydroxyl radical with dimethyl methylphosphonate, DMMP, (reaction (1)) was studied using direct techniques by two groups using two different methods of generation of hydroxyl radicals over an extended temperature range of 273–837 K.

The room temperature rate constant is in reasonable agreement with the only previous indirect determination [17].

A V-shaped temperature dependence, negative at low temperatures, and positive at higher temperatures, with a turning point at 530 ± 10 K, was unambiguously established.

Significant dependence of the rate constant on the bath gas pressure (He) was observed. The observations are consistent with the assumption that the major channel of this reaction is abstraction of H-atom from methoxy groups, in which the reaction barrier lies below the ground state of the reactants.

This study is the first direct study of the title reaction. Wide temperature range allowed revealing the peculiar V-shaped temperature dependence. This is the second reaction of hydroxyl radicals with an organophosphorus compound that exhibits such behavior.

It is apparent that extended experimental (including bath gas pressure dependence over extended pressure and temperature ranges) as well at theoretical studies of this and other elementary reactions of this class, are required for better understanding of the mechanism of these reactions.

## Figures and Tables

**Table 1:** Experimental conditions for the experiments with O_3_/O_2_/H_2_O/DMMP/He + 266 nm. All measurements at 1.00 ± 0.02 bar.

Temperature/K	[O_3_]/10^14^ Molecule cm^−3^	[O_2_]/10^15^ Molecule cm^−3^	[H_2_O]/10^16^ Molecule cm^−3^	[He]/10^19^ Molecule cm^−3^	[DMMP]/10^14^ Molecule cm^−3^	k’/10^3^s^−1^	k/10^−12^ cm^3^ Molecule^−1^ s^−1^
299	4.57	9.80	3.38	2.44	0	0.467	10.5
299	4.57	9.80	3.38	2.44	4.70	5.03	
299	4.57	9.80	3.38	2.44	9.41	9.98	
299	4.57	9.77	3.37	2.44	14.1	15.2	
299	4.57	9.75	3.37	2.44	18.73	19.9	
299	4.57	9.68	3.34	2.44	23.2	25.4	
299	4.54	9.69	3.42	2.44	0	0.511	9.85
299	4.54	9.62	3.4	2.44	4.73	5.55	
299	4.54	9.46	3.38	2.44	9.42	9.83	
299	4.54	9.32	3.33	2.44	13.9	14.3	
299	4.54	10.31	3.51	2.44	19.6	19.1	
299	4.54	10.35	3.49	2.44	24.3	23.9	
328	2.78	9.05	3.30	2.26	0	0.450	6.70
328	2.78	8.79	3.14	2.26	4.37	3.22	
328	2.78	8.66	3.10	2.26	8.61	6.25	
328	2.78	8.51	3.08	2.26	12.8	8.33	
328	2.78	8.47	3.07	2.26	17.0	13.2	
328	2.78	8.29	3.04	2.26	21.1	14.2	
365	1.78	8.19	2.77	2.02	0	0.452	4.75
365	1.78	8.18	2.74	2.02	3.97	1.95	
365	1.78	8.22	2.67	2.02	7.98	4.16	
365	1.78	8.08	2.65	2.02	11.9	5.88	
365	1.78	8.03	2.63	2.02	15.7	7.69	
365	1.78	7.83	2.60	2.02	19.4	8.33	
365	3.97	8.15	2.57	2.00	0	0.452	5.06
365	3.97	7.99	2.63	2.00	4.02	2.32	
365	3.97	7.95	2.62	2.00	8.00	4.16	
365	3.97	7.84	2.61	2.00	11.9	6.66	
365	3.97	7.70	2.60	2.00	15.8	8.33	
365	3.97	7.53	2.62	2.00	19.6	9.09	
365	3.97	8.72	2.67	2.00	0	0.413	5.04
365	3.97	8.63	2.64	2.00	12.4	6.66	
365	3.97	8.46	2.62	2.00	16.4	9.09	
365	3.97	8.30	2.60	2.00	20.4	11.1	
365	3.97	8.16	2.58	2.00	24.3	12.5	
365	3.97	7.93	2.54	2.00	11.9	6.66	
365	3.97	7.79	2.53	2.00	23.8	12.7	
411	3.44	7.50	2.31	1.80	0	0.450	3.99
411	3.44	7.64	2.30	1.80	3.67	1.75	
411	3.44	7.34	2.29	1.80	7.30	3.03	
411	3.44	7.20	2.27	1.80	10.8	4.54	
411	3.44	7.06	2.25	1.80	14.3	6.25	
411	3.44	7.02	2.24	1.80	17.8	7.14	
470	1.88	6.44	1.99	1.59	0	0.478	3.16
470	1.88	6.39	1.99	1.59	3.22	1.38	
470	1.88	6.28	1.99	1.59	6.40	2.22	
470	1.88	6.56	1.98	1.59	9.55	3.33	
470	1.88	5.39	1.96	1.58	12.6	4.16	
470	1.88	6.23	1.95	1.59	15.6	5.55	

**Table 2:** Experimental conditions for the experiments with N_2_O/H_2_O/DMMP + 193.3 nm. All measurements at 1.00 ± 0.02 bar (He).

Temperature/K	[H_2_O]/10^17^ Molecule cm^−3^	[N_2_O]/10^16^ Molecule cm^−3^	[DMMP]/10^14^ Molecule cm^−3^	k’/10^3^ s^−1^	k/10^−12^ cm^3^ Molecule ^−1^ s^−1^
295	3.03	3.23	0	0.847	8.51
295	3.03	3.23	10.0	9.25	
295	3.03	3.23	8.0	7.57	
295	3.03	3.23	6.0	5.95	
295	3.03	3.23	4.0	4.21	
295	3.03	3.23	2.0	2.24	
295	3.03	3.23	0	0.794	
323	2.98	7.70	0	0.448	6.82
323	2.98	7.70	7.56	5.61	
323	2.98	7.70	6.05	4.60	
323	2.98	7.70	4.54	3.43	
323	2.98	7.70	3.02	2.34	
323	2.98	7.70	1.51	1.51	
323	2.98	7.70	0	0.442	
365	2.91	4.49	0	0.463	5.36
365	2.91	4.49	9.59	5.68	
365	2.91	4.49	7.68	4.58	
365	2.91	4.49	5.76	3.71	
365	2.91	4.49	3.84	2.77	
365	2.91	4.49	1.92	1.60	
411	3.03	5.99	0	0.360	4.17
411	3.03	5.99	10.0	4.566	
411	3.03	5.99	8.0	3.77	
411	3.03	5.99	6.0	3.04	
411	3.03	5.99	4.0	2.24	
411	3.03	5.99	2.0	1.324	
411	3.03	5.99	0	0.465	
455	3.24	5.44	0	0.341	3.59
455	3.24	5.44	9.62	3.83	
455	3.24	5.44	7.70	3.09	
455	3.24	5.44	5.77	2.52	
455	3.24	5.44	3.85	1.89	
455	3.24	5.44	1.92	1.05	
455	3.24	5.44	0	0.394	
500	2.81	4.23	0	0.371	3.16
500	2.81	4.23	9.27	3.26	
500	2.81	4.23	7.42	2.74	
500	2.81	4.23	5.56	2.16	
500	2.81	4.23	3.71	1.48	
500	2.81	4.23	1.85	1.00	
500	2.81	4.23	0	0.350	
540	2.46	3.64	0	0.34	2.98
540	2.46	3.64	9.02	3.08	
540	2.46	3.64	7.22	2.51	
540	2.46	3.64	5.41	1.98	
540	2.46	3.64	3.61	1.51	
540	2.46	3.64	1.80	1.03	
540	2.46	3.64	0	0.353	
591	2.73	4.17	0	0.388	3.17
591	2.73	4.17	9.82	3.50	
591	2.73	4.17	7.86	2.91	
591	2.73	4.17	5.90	2.27	
591	2.73	4.17	3.93	1.70	
591	2.73	4.17	1.96	1.03	
591	2.73	4.17	0	0.427	
673	2.42	4.85	0	0.355	3.85
673	2.42	4.85	8.70	3.65	
673	2.42	4.85	6.96	3.05	
673	2.42	4.85	5.22	2.51	
673	2.42	4.85	3.48	1.74	
673	2.42	4.85	1.74	1.06	
673	2.42	4.85	0	0.346	
773	2.10	3.55	0	0.424	5.16
773	2.10	3.55	7.60	4.34	
773	2.10	3.55	6.06	3.57	
773	2.10	3.55	4.54	2.84	
773	2.10	3.55	3.03	2.14	
773	2.10	3.55	1.51	1.33	
773	2.10	3.55	0	0.412	
837	1.94	3.27	0	0.465	6.07
837	1.94	3.27	9.89	6.45	
837	1.94	3.27	7.91	5.31	
837	1.94	3.27	5.94	4.18	
837	1.94	3.27	3.96	3.01	
837	1.94	3.27	1.98	1.90	
837	1.94	3.27	0	0.385	
